# Refractory Short-Lasting Unilateral Neuralgiform Headache Attacks With Conjunctival Injection and Tearing (SUNCT) Responding to Erenumab Adjuvant Therapy: A Case Report

**DOI:** 10.7759/cureus.24403

**Published:** 2022-04-23

**Authors:** Vishali Moond, Katherine Hamilton, Rebecca Martinez, Claudia Carrizo, Mark Burish

**Affiliations:** 1 Internal Medicine, University College of Medical Sciences, Delhi, IND; 2 Department of Neurology, Georgetown University Medical Center, Washington DC, USA; 3 Department of Neurosurgery, University of Texas Health Science Center at Houston, Houston, USA

**Keywords:** sunct, short-lasting unilateral neuralgiform headache attacks, sunha, erenumab, calcitonin gene related peptide

## Abstract

Many patients with short-lasting unilateral neuralgiform headache attacks with conjunctival injection and tearing (SUNCT) fail to respond to the first-line treatment of lamotrigine. Additionally, data for other treatments are limited in this rare headache disorder. SUNCT involves activation of the trigeminal nerve which uses the neuropeptide calcitonin gene-related peptide (CGRP); thus CGRP-targeted treatments may be beneficial in this disorder. We present a patient with SUNCT who failed to respond optimally to 10 medications and four surgical treatments. However, she had minimal attacks after erenumab 140 mg was added to carbamazepine 200 mg three times daily and pregabalin 75 mg twice daily. Decreasing any of these three medications worsened her attacks. Our case represents the second case report of a SUNCT patient responding to a CGRP monoclonal antibody, suggesting this treatment may be a consideration in refractory SUNCT.

## Introduction

The trigeminal autonomic cephalalgias (TACs) are a group of primary headache disorders characterized by severe unilateral headaches accompanied by either ipsilateral cranial autonomic features, restlessness, or both [1]. The TACs consist of five headache disorders: cluster headache, paroxysmal hemicrania, short-lasting unilateral neuralgiform headache attacks with conjunctival injection and tearing (SUNCT), short-lasting unilateral neuralgiform headache attacks with cranial autonomic symptoms (SUNA), and hemicrania continua. These conditions differ in their attack duration, attack frequency, and treatments [2]. SUNCT, the disease discussed in this case, is officially characterized by 1) at least 20 attacks, 2) moderate or severe pain in the temple and/or one or more distributions of the trigeminal nerve lasting between 1 second and 10 minutes, 3) ipsilateral autonomic features which must include both conjunctival injection and lacrimation, 4) at least one attack most days unless in remission, and 5) not better accounted for by another diagnosis [1]. 

Data on treatments for SUNCT are limited to our knowledge, there is currently only one randomized placebo-controlled clinical trial of SUNCT, an underpowered study of topiramate [3]. Based on open-label studies and expert opinion, the first-line treatment is lamotrigine [4]. Intravenous lidocaine may be the most effective treatment [2,5], however, administration requires experienced providers and specialized monitoring given IV lidocaine’s risk of cardiac arrhythmias. Other treatments include carbamazepine, oxcarbazepine, topiramate, gabapentin, pregabalin, duloxetine, and mexiletine [2,6], and case reports have suggested the effectiveness of microvascular decompression [7]. Unfortunately, patients often do not respond to these treatments.

A recent case report of SUNCT noted the effectiveness of monoclonal antibodies against calcitonin gene-related peptide (CGRP) and the CGRP receptor [8]. Here we provide the second case of a SUNCT patient who responded to a CGRP receptor monoclonal antibody. In our case, the patient had refractory SUNCT and responded to erenumab as an adjunctive treatment. The patient consented to publish her case and edited the final version of the manuscript prior to submission.

## Case presentation

A 67-year-old woman with past history notable for mitral valve disease status post repair presented with pain in the right orbital, periorbital, frontal, and upper maxillary areas that started at the age of 51. The attacks lasted about 30 seconds and occurred between 20 and 100 times daily. She was typically pain-free in between attacks though could have some inter-ictal pain; it was difficult to discern if her occasional inter-ictal pain was the classic saw-tooth pain seen in some SUNCT patients. The attacks were sharp, stabbing, and had an intensity of 10 on the 0-10 numerical rating scale. These attacks were accompanied by ipsilateral conjunctival injection, lacrimation, nasal congestion, and ptosis, as well as bilateral rhinorrhea, photophobia, and nausea. The attacks could occur spontaneously or be triggered by wind hitting her face, touching her face near the pain, chewing, washing her face, brushing her teeth, crying, coughing, sneezing, or swallowing. Diagnostic considerations for this patient over her 16-year headache course included both SUNCT and trigeminal neuralgia. Her work-up and management targeted one or both disorders as detailed below. 

Diagnostic assessment and treatment trials

A brain MRI with and without contrast in 2019 was unremarkable as was a chest X-ray. Laboratory examinations were unremarkable including a standard chemistry panel, liver function tests, and a hormone panel (specifically thyroid-stimulating hormone, adrenocorticotropic hormone, growth hormone, prolactin, cortisol, luteinizing hormone, follicle-stimulating hormone, testosterone, and estrogen). Physical examination was unremarkable except for decreased sensation to light touch over the right forehead and cheek attributed to post-surgical changes following a rhizotomy.

She tried a variety of treatments through multiple providers, which are summarized in Table 1. Indomethacin 75mg three times daily for two weeks was ineffective, thus ruling out paroxysmal hemicrania and other indomethacin-responsive headaches. Multiple sodium channel blockers, which are often effective in SUNCT and trigeminal neuralgia (carbamazepine, oxcarbazepine, and lamotrigine) were insufficient. Other medications for trigeminal neuralgia (baclofen and clonazepam) were similarly ineffective, as were more general neuropathic pain medications (gabapentin, pregabalin, topiramate, amitriptyline, and tramadol). 

**Table 1:** Treatment trials for our case. We consider our patient’s SUNCT to be refractory as she failed to respond to multiple recommended medications.

Treatment	Maximum dose	Typical indication for this treatment	Result
Amitriptyline	40 mg at bedtime	Neuropathic pain (multiple types), migraine	No effect, daytime sleepiness
Baclofen	Unknown	Trigeminal neuralgia, cluster headache, muscular pain	No effect
Carbamazepine	200 mg in the morning, 200 mg in the afternoon, 400 mg at bed	Trigeminal neuralgia, SUNCT	No effect until erenumab was added
Clonazepam	0.5 mg twice daily	Trigeminal neuralgia	No effect
Erenumab	140 mg	Migraine	No effect at 70 mg for two months, effective at 140 mg in combination with other medications
Gabapentin	Unknown	Neuropathic pain (multiple types)	No effect
Indomethacin	75 mg three times daily for two weeks	Paroxysmal hemicrania	No effect
Lamotrigine	50 mg twice daily	Trigeminal neuralgia, SUNCT	No effect, rash
Oxcarbazepine	900 mg twice daily	Trigeminal neuralgia, SUNCT	No effect, hyponatremia
Pregabalin	75 mg twice daily	Neuropathic pain (multiple types)	No effect until erenumab was added
Topiramate	Unknown	Neuropathic pain, migraine, cluster headache	No effect
Tramadol	Unknown	Multiple pain conditions	No effect

Multiple surgical treatments for trigeminal neuralgia provided only temporary relief including stereotactic radiosurgery, microvascular decompression, and rhizotomy, which are summarized in Table 2.

**Table 2:** Procedures undergone by the patient. The patient received temporary or no relief from four different surgical treatments, some repeated multiple times, though it is difficult to claim these procedures imply that she is refractory:  these procedures are primarily used for trigeminal neuralgia and their data in SUNCT is limited.

Procedures	Number of times performed	Typical indication for this treatment	Result
Stereotactic radiosurgery	3	Trigeminal neuralgia	a few months of relief each time
Microvascular decompression	1	Trigeminal neuralgia	1.5 years of relief, then the pain returned
Rhizotomy	2	Trigeminal neuralgia	1 year of relief the first time, no effect the second time
Trigeminal nerve stimulation	1	Neuropathic pain (multiple types)	no effect, stimulator removed

In March 2020, the patient found no relief with a combination of lamotrigine 50 mg twice daily (and was titrating down due to potential rash), pregabalin 50 mg three times daily, and carbamazepine 200 mg in the morning, 200 mg in the afternoon, and 400 mg at night. Additionally, this combination of therapies was causing sedation. Erenumab was started at 70 mg monthly for two months, which had no effect. The dose was increased to 140 mg monthly, and the patient noticed a dramatic reduction in the number of daily attacks for four days after the first injection, then a sustained reduction in attacks after subsequent injections, specifically a noticeable decrease in the number of daily attacks, the intensity of each attack, and an improved ability to talk without triggering pain. The patient stopped lamotrigine and was able to down titrate carbamazepine. However, the regimen of erenumab 140 mg monthly, carbamazepine 200 mg three times daily, and pregabalin 75 mg twice daily appeared to be the minimum required regimen: any reductions below these doses caused worsened pain. The patient has been on this regimen since July 2021; as of the last contact with the patient on March 24, 2022, she was having 1-5 attacks per day of average 1 out of 10 pain intensity with no cutaneous triggers, all within the first hour of awakening (though they do not cause her to wake up). She reports no sedation, constipation, or other side effects on this regimen. Her last laboratory evaluation in August 2021 showed a normal complete blood count (CBC), sodium level, and liver function tests. Of note, she had lung cancer diagnosed in December 2021, discovered after developing a cough in October 2021. According to the patient, her biopsy revealed stage 3b non-small cell lung cancer, and additional imaging showed no metastases. We suspect that her lung cancer is unrelated to her headaches given the onset of her headaches (at age 51) in relation to the onset of her pulmonary symptoms (at age 69) as well as the lack of metastasis.

## Discussion

SUNCT is a rare disorder, with a prevalence of 6.6 per 100,000 and an annual incidence of 1.2 per 100,000 people [5]. The typical onset is over the age of 40 years [9]. Mimics of SUNCT include primary headaches with similar features and secondary headaches with SUNCT-like features. For primary headaches, SUNCT can be confused for paroxysmal hemicrania, which has unilateral pain and ipsilateral cranial autonomic features and overlaps in duration with SUNCT (paroxysmal hemicrania attacks last 2-30 minutes, while SUNCT attacks last between 1 second and 10 minutes). However paroxysmal hemicrania is exquisitely sensitive to indomethacin while SUNCT is not. SUNCT can also be confused with trigeminal neuralgia as they share a similar duration, location, and cutaneous triggers over the area of pain. However, unlike trigeminal neuralgia, SUNCT has strong ipsilateral cranial autonomic features and no refractory period after cutaneous triggering [2]. For secondary headaches, several systematic reviews of case series have identified causes of SUNCT-like headaches including pituitary tumors (the most common cause), other neoplasms (carcinoid, epidermoid, meningioma, pilocytic astrocytoma, lung adenocarcinoma), vascular (aneurysm, cavernous sinus dural fistula, compression of the trigeminal nerve by the superior or anterior inferior cerebellar artery), inflammatory/infectious (multiple sclerosis, viral meningitis), or an orbital cyst [7,10]. In these cases, treatment of the underlying condition resolved the headaches. Because of these secondary headaches, recommended imaging for all SUNCT patients includes a brain MRI; however, in refractory cases, providers should consider pituitary function testing, imaging of the apex of the lung, vessel imaging of the head and neck, and a high-resolution MRI of the brainstem [11]. Our patient met all official criteria [1] for SUNCT, failed to respond to indomethacin, and had a negative workup for secondary headaches including a normal MRI, normal chest X-ray, and normal pituitary hormone testing. A magnetic resonance angiography (MRA) was not performed to our knowledge, but the patient did have a microvascular decompression which was only temporarily helpful.

The etiology and pathogenesis of SUNCT are unknown but the trigeminal nerve appears to be involved, as cutaneous triggers in the trigeminal nerve distribution are found in 79% of patients [12]. The trigeminal nerve uses multiple pain neuropeptides including calcitonin gene-related peptide (CGRP). CGRP and CGRP receptor monoclonal antibodies are effective in migraine [13] and one is effective in cluster headaches [14]; both of these disorders involve trigeminal nerve pain pathways [15]. Thus, other disorders involving the trigeminal nerve, such as SUNCT, may also respond to CGRP and CGRP receptor antibodies. A recent case report described erenumab as an effective treatment for a patient with SUNCT [8]. Our case is similar to the prior case report in that our patient was the same age (67 years old) and was refractory to treatments including indomethacin, lamotrigine, gabapentin, and topiramate. In both cases, erenumab monotherapy was insufficient in preventing attacks; in our case, it consistently reduced the daily attacks, while in the published case it consistently eliminated the attacks only for two-three weeks. We found that erenumab 140 mg was highly effective when combined with a low dose of a sodium channel blocker (carbamazepine) and a moderate dose of neuropathic pain medication (pregabalin), while in the published case the patient was changed to galcanezumab monotherapy with excellent results.

Strengths of this case report include the trial of multiple treatments before trying erenumab, suggesting that the patient was truly refractory. Weaknesses include the fact that this is a single case report, and it is difficult to know what true combination of CGRP antibody, sodium channel blocker, and gabapentinoid is necessary: we did not try other CGRP antibodies, and after finding the minimum needed dose of each medication, it was decided not to further alter an effective regimen with few side effects.

## Conclusions

Our case identifies a patient with refractory SUNCT who responded to erenumab 140 mg monthly when added to low-dose carbamazepine and moderate-dose pregabalin. We think that additional studies of CGRP monoclonal antibodies are warranted for this disorder given the connections between the CGRP system, the trigeminal nerve, and SUNCT, as well as the existence of two case reports on the benefits of CGRP monoclonal antibodies in SUNCT.
